# Epidemiological characteristics of childhood infectious diseases at Qingyang maternal and child health care hospital during the COVID-19 pandemic and the post-pandemic period

**DOI:** 10.3389/fped.2026.1826285

**Published:** 2026-06-01

**Authors:** Xiangfeng Qi, Yulin Feng, Beiqin Liu, Yuelong Hui

**Affiliations:** 1 Qingyang Maternal and Child Health Care Hospital, Qingyang, Gansu, China; 2 Qing Yang Municipal Center for Disease Control and Prevention, Qingyang, Gansu, China

**Keywords:** analysis, childhood infectious diseases, coronavirus disease 2019 (COVID-19) pandemic periods, epidemiological characteristics, post-pandemic periods

## Abstract

**Objective:**

To characterize the epidemiological patterns of childhood infectious diseases during and after the COVID-19 pandemic and to inform optimization of pediatric prevention and control strategies.

**Methods:**

A descriptive epidemiological analysis was conducted on reported infectious disease cases among children aged ≤14 years at Qingyang Maternal and Child Health Care Hospital. The pandemic period was defined as 2020–2022, and the post-pandemic period as 2023–2025. Proportions were compared using the chi-square test.

**Results:**

From 2020 to 2025, a total of 2,559 cases were reported, yielding an overall reporting rate of 5.28 per 1,000 outpatient visits. Only 53 cases (0.42‰) occurred during the pandemic period, whereas 2,506 cases (6.98‰) occurred post-pandemic. The annual reporting rate ranged from 0.37‰ in 2022 to 10.32‰ in 2025. The male-to-female ratio was 1.30:1. The proportion of cases aged ≤2 years increased from 33.96% during the pandemic to 46.89% post-pandemic, while the 7–14 year age group declined from 28.30% to 15.96%. Nearly all cases (98.94%) resided in Qingyang City. The disease spectrum shifted markedly: varicella (45.28%), pertussis (22.64%), and mumps (11.32%) predominated during the pandemic, whereas influenza (48.72%), other infectious diarrheal diseases (26.94%), and hand-foot-mouth disease (12.01%) dominated the post-pandemic period. Case counts for pertussis, diarrheal diseases, hand-foot-mouth disease, and varicella increased significantly across multiple age strata post-pandemic. Total medical expenditures reached ¥5,623,550.62, with 98.70% incurred post-pandemic; influenza [¥2,746,750.98 (RMB)] and other infectious diarrheal [¥2,455,076.20 (RMB)] constitute the primary economic burden.

**Conclusion:**

The pediatric infectious disease spectrum underwent a substantial post-pandemic transformation, with influenza, diarrheal diseases, and hand-foot-mouth disease replacing varicella and pertussis as dominant conditions. Children aged ≤6 years emerged as the primary affected population. The high caseload and economic burden of influenza and diarrheal diseases underscore the need for strengthened surveillance, prioritized protection of younger children, optimized resource allocation, and refined vaccination strategies in the evolving epidemiological landscape.

## 1 Introduction

The coronavirus disease 2019 (COVID-19) pandemic, as a major global public health emergency (1, 2), has profoundly reshaped societal operations and population behavioral patterns (3, 4), while also exerting significant impacts on health care systems—particularly on the disease spectrum and disease burden observed in specialty institutions. To curb the transmission of SARS-CoV-2, the World Health Organization and governments worldwide implemented a range of measures, including widespread COVID-19 vaccination (5), strengthening surveillance networks, upgrading laboratory diagnostic capacity to enhance viral detection, promoting the use of medical masks, encouraging frequent hand hygiene, ensuring adequate indoor ventilation, and advising the public to minimize non-essential outings or maintain a physical distance of at least 1 meter (3 feet) (6). In China, stringent non-pharmaceutical interventions (NPIs) were enforced (7, 8), Under these NPIs, the epidemiological patterns of other common respiratory infectious diseases—including pertussis, measles, scarlet fever, tuberculosis, mumps, and influenza—were substantially altered (9, 10), Moreover, changes in human mobility further influenced the transmission dynamics of these infections (11). On January 8, 2023, China adjusted its COVID-19 control strategy, reclassifying the disease from “Class A management for Class B infectious diseases” to “Class B management for Class B infectious diseases” (“Category B, managed as Category B”). Consequently, to inform evidence-based prevention and control strategies for emerging and re-emerging infectious diseases in the post-pandemic era, it is essential to systematically examine the broad impact of NPIs—not only during the pandemic but also in its aftermath—on non-COVID-19 infectious diseases. Notably, research specifically focusing on maternal and child health care institutions remains relatively scarce.

In advancing China's Sustainable Development Goals, maternal and child health hospitals have played a pivotal role by delivering efficient, high-quality services (12). The epidemiological characteristics of infectious diseases among pediatric patients attending such institutions directly reflect shifts in disease risk within this vulnerable population before, during, and after major public health emergencies. Although national-level maternal and child health service delivery in China compares favorably with that in most other countries (13), substantial regional disparities persist (14), underscoring the need for more localized, subnational investigations. Furthermore, since the nationwide implementation of the universal two-child policy in 2015, and the subsequent encouragement of families to have up to three children by 2021 (15), demand for maternal and child health services has continued to rise, necessitating optimized allocation of health resources. This study aims to characterize the epidemiological features of common childhood infectious diseases at the Qingyang Maternal and Child Health Hospital during and after the COVID-19 pandemic. Findings will provide empirical evidence and decision-support insights to guide the optimization of triage protocols and targeted infection prevention and control strategies for children in maternal and child health institutions in the post-pandemic era.

## 2 Study population and methods

### 2.1 Overview

Qingyang Maternal and Child Health Care Hospital (Qingyang Women's and Children's Hospital), established in 1963, has a 60-year history of service. It is designated by the Gansu Provincial Health Commission as a Grade III–B maternal and child health care institution. The hospital provides comprehensive medical services to children across all counties and districts of Qingyang City, as well as to pediatric populations from neighboring cities. It maintains 500 fixed inpatient beds and operates 27 clinical and medical technology departments. Annually, it serves over 100,000 outpatient visits and discharges approximately 10,000 inpatients. As the municipal center for maternal and child health care guidance, the hospital integrates clinical care, preventive health services, reproductive health technical support, health education, public health management for women and children, and teaching and scientific research.

### 2.2 Study population and data sources

We collected infectious disease surveillance data for children aged ≤14 years, reported by Qingyang Maternal and Child Health Care Hospital to the China Information System for Disease Control and Prevention (CISDCP) during the COVID-19 pandemic period (2020–2022) and the post-pandemic period (2023–2025). Pediatric outpatient, emergency, and inpatient visit records were extracted from the hospital's outpatient and electronic medical record databases. Infectious disease diagnoses were classified according to the International Classification of Diseases, Tenth Revision (ICD-10). The CISDCP database contains all nationally mandated infectious disease reports since the implementation of China's national online direct reporting system in 2004, including variables such as case count, age group, gender, and mortality rates.

### 2.3 Inclusion criteria and age group definition

The analysis included the 40 notifiable infectious diseases stipulated by Chinese law, as well as other managed and key surveillance infectious diseases; only cases among children aged ≤14 years were included. The primary age stratification—comprising three groups (<1 year, 1–6 years, and 7–14 years)—was established based on principles of developmental immunology and differences in exposure risk associated with collective settings among children. For certain diseases, applying this primary age framework would obscure critical epidemiological or programmatic characteristics; therefore, refined age cutoffs were adopted. For mumps, a threshold of 2 years was selected to align with the timing of the second dose of the measles-mumps-rubella (MMR) vaccine, which is routinely administered at 18 months of age. This distinction facilitates clear differentiation between pre-vaccination susceptibility and post-vaccination immunity. For scarlet fever, incidence peaks sharply during early school age (3–10 years), whereas the disease is exceedingly rare in infancy. To accurately characterize the age-specific disease burden, stratification was refined into three categories: ≤2 years, 3–10 years, and 11–14 years.

### 2.4 Study methods

This study employed descriptive epidemiological methods to comparatively analyze the distribution of pediatric infectious disease cases across classifications including gender, age group, geographic origin (county/district), disease diagnosis, and reporting date. Data entry and organization were completed using Microsoft Excel 2010, while statistical analysis was performed with SPSS 20.0 software. Categorical variables were presented as percentages; intergroup comparisons of proportions were conducted using the chi-square test. Additionally, to evaluate the temporal trend in reported incidence rates from 2020 to 2025, Poisson regression was used with calendar year modeled as a continuous variable and the natural logarithm of outpatient visit volume (in thousands) included as an offset. The Cochran-Armitage test for trend in proportions was also performed to assess the consistency of the trend. A two-sided *P*-value <0.05 was considered statistically significant, with the significance level set at *α* = 0.05. The infectious disease reporting rate was calculated as: number of reported pediatric infectious disease cases divided by the total number of pediatric outpatient, emergency, and inpatient visits during the same period. USD amount = RMB amount/USD–CNY exchange rate.

### 2.5 Ethical approval

This study was approved by the Medical Ethics Committee of Qingyang Maternal and Child Health Care Hospital, Gansu Province [Ethics Approval No.: QFY-LW (2026) 001].

## 3 Results

### 3.1 Infectious disease reporting status

From 2020 to 2025, a total of 2,559 infectious disease cases were reported among children aged 0–14 years at this hospital, yielding an overall reporting rate of 5.28 per thousand. Between 2020 and 2022, only 53 cases were reported, corresponding to a reporting rate of 0.42 per thousand; in contrast, from 2023 to 2025, 2,506 cases were reported, with a markedly higher reporting rate of 6.98 per thousand. The highest annual reporting rate was observed in 2025 (10.32 per thousand), whereas the lowest occurred in 2022 (0.37 per thousand). See Table 1. The temporal trend in reported incidence rates from 2020 to 2025 was assessed using Poisson regression with calendar year modeled as a continuous variable and the natural logarithm of outpatient visit volume (in thousands) included as an offset. A statistically significant increasing trend was observed (*β* = 0.55, *P* < 0.001), equivalent to an average annual increase of 74% in incidence (IRR = 1.74, 95% CI: 1.63–1.85). The Cochran-Armitage test for trend in proportions yielded consistent results (*χ*^2^ = 1,302.2, *P* < 0.001).

**Table 1:** Analysis of the reporting rate of children's infectious diseases at Qingyang maternal and child health hospital (Qingyang women and children's hospital) from 2020 to 2025.

Year	Number of outpatient visits	Number of reported cases	Reported rate of infectious diseases (per thousand)
2020	17,232	8	0.46
2021	19,594	12	0.61
2022	89,350	33	0.37
2023	1,18,591	466	3.93
2024	1,16,424	762	6.55
2025	1,23,817	1,278	10.32
Total	4,85,008	2,559	5.28

### 3.2 General characteristics of reported pediatric infectious disease cases

Among the 2,559 pediatric infectious disease cases reported from 2020 to 2025, 1,447 (56.6%) were male and 1,112 (43.4%) were female, yielding a male-to-female sex ratio of 1.30:1. The proportion of male cases was significantly higher than that of female cases (*χ*^2^ = 87.112, *P* < 0.05). Notably, the proportion of male cases declined significantly between the 2023–2025 period compared with the 2020–2022 period (*χ*^2^ = 432.082, *P* < 0.05), whereas the proportion of female cases increased significantly over the same interval (*χ*^2^ = 334.634, *P* < 0.05).

Regarding age distribution, among cases reported from 2020 to 2022, children aged ≤2 years accounted for 33.96%, those aged 3–6 years for 37.74%, and those aged 7–14 years for 28.30%. In contrast, among cases reported from 2023 to 2025, the corresponding proportions were 46.89%, 37.15%, and 15.96%, respectively. The difference in age-group distribution across the two time periods was statistically significant (*χ*^2^ = 373.721, *P* < 0.05).

Geographically, locally resident cases (i.e., cases from the city under study) constituted 98.94% (2,532/2,559) of all reported cases. Among the subset of cases with complete geographic information (*n* = 2,506), locally resident cases accounted for 98.96% (2,480/2,506). The distribution of cases across different geographic regions differed significantly (*χ*^2^ = 124.005, *P* < 0.05) (See Table 2).

**Table 2:** Basic analysis of children's infectious diseases in Qingyang maternal and child health hospital (Qingyang women and children's hospital) from 2020 to 2025.

Demographic characteristics	2020–2022		2023–2025		Total		*χ* ^2^	*P*
	Reported cases	Proportion (%)	Reported cases	Proportion (%)	Reported cases	Proportion (%)		
Sex								
Male	31	58.49	1,416	56.50	1,447	56.55	432.082	0.000
Female	22	41.51	1,090	43.50	1,112	43.45	334.634	0.000
Age (years)								
<1 year	13	24.53	344	13.73	357	13.95	60.071	0.000
1–2 years	5	9.43	831	33.16	836	32.67	289.247	0.000
3–6 years	20	37.74	931	37.15	951	37.16	269.553	0.000
7–14 years	15	28.30	400	15.96	415	16.22	159.062	0.000
Residence								
County/district of residence	42	79.25	1,816	72.47	1,858	72.61	669.585	0.000
Other counties/districts within the same city	10	18.87	664	26.50	674	26.34	149.838	0.000
Other prefecture-level cities within the same province	0		10	0.40	10	0.39	0.068	0.794
Counties/districts outside the province	1	1.89	16	0.64	17	0.66	0.004	0.947

### 3.3 Disease spectrum analysis of infectious diseases in children

From 2020 to 2022, the top three reported infectious diseases among children were varicella (45.28%), pertussis (22.64%), and mumps (11.32%). From 2023 to 2025, the leading reported infectious diseases shifted to influenza (48.72%), other infectious diarrheal diseases (26.94%), and hand-foot-mouth disease (12.01%). The differences in case distribution across disease categories were statistically significant (*χ*^2^ = 6502.157, *P* < 0.05). Compared with the pandemic period (2020–2022), the proportions of reported cases of varicella, pertussis, and mumps declined significantly during the post-pandemic period (2023–2025) (*χ*^2^ = 45.762, *P* < 0.05; *χ*^2^ = 17.498, *P* < 0.05; *χ*^2^ = 21.889, *P* < 0.05, respectively). Conversely, the proportions of reported cases of influenza, other infectious diarrheal diseases, and hand-foot-mouth disease all increased significantly (*χ*^2^ = 583.298, *P* < 0.05; *χ*^2^ = 313.917, *P* < 0.05; *χ*^2^ = 140.695, *P* < 0.05, respectively). Congenital syphilis was reported in three cases during the pandemic period but showed zero reported cases in the post-pandemic period (See Table 3).

**Table 3:** Analysis of the disease spectrum of pediatric infectious diseases at Qingyang maternal and child health hospital (Qingyang women and children's hospital) from 2020 to 2025.

Disease names	2020–2022			2023–2025			Total			*χ* ^2^	*P*
	Reported cases	Proportion (%)	Infectious disease reporting rate (‰)	Reported cases	Proportion (%)	Infectious disease reporting rate (‰)	Reported cases	Proportion (%)	Infectious disease reporting rate (‰)		
Influenza	0	0.00	0.00	1,224	48.84	3.89	1,224	47.83	2.64	583.298	0.000
Other infectious diarrheal diseases	3	5.66	0.02	675	26.94	2.14	678	26.49	1.46	313.917	0.000
Hand, foot, and mouth disease	1	1.89	0.01	301	12.01	0.96	302	11.80	0.65	140.695	0.000
Varicella	24	45.28	0.16	196	7.82	0.62	220	8.60	0.47	45.762	0.000
Pertussis	12	22.64	0.08	85	3.39	0.27	97	3.79	0.21	17.498	0.000
Mumps	6	11.32	0.04	5	0.20	0.02	11	0.43	0.02	1.593	0.207
Scarlet fever	1	1.89	0.01	10	0.40	0.03	11	0.43	0.02	1.739	0.187
Congenital syphilis	3	5.66	0.02	0	0.00	0.00	3	0.12	0.01	3.590	0.058
Hepatitis A	0	0.00	0.00	2	0.08	0.01	2	0.08	0.00	0.048	0.827
Measles	0	0.00	0.00	1	0.04	0.00	1	0.04	0.00	0.000	1.000
Other diseases	3	5.66	0.02	7	0.28	0.02	10	0.39	0.02	0.000	1.000

**Table 4:** Age-stratified analysis of pediatric infectious disease cases at Qingyang maternal and child health hospital (Qingyang municipal women and children's hospital) from 2020 to 2025.

Disease name	Age group	2020–2022		2023–2025		*χ* ^2^	*P*
		Reported cases	Proportion (%)	Reported cases	Proportion (%)		
Influenza	<1 years	0	–	124	10.16	31.098	0.000
	1–6 years	0	–	854	69.94	294.693	0.000
	7–14 years	0	–	243	19.90	117.658	0.000
Pertussis	<1 years	7	58.33	23	27.06	0.204	0.651
	1–6 years	4	33.33	33	38.82	4.246	0.039
	7–14 years	1	8.33	29	34.12	11.683	0.001
Other infectious diarrheal	<1 years	1	33.33	175	25.96	41.691	0.000
	1–6 years	2	66.67	496	73.59	166.269	0.000
	7–14 years	0	0.00	3	0.45		0.556^a^
Mumps	<2 years	0	0.00	0	0.00	/	/
	≥2 years	6	100.00	5	100.00	2.678	0.102
Hand, foot, and mouth disease	<1 years	0	0.00	12	3.99	1.886	0.170
	1–6 years	1	100.00	254	84.39	85.124	0.000
	7–14 years	0	0.00	35	11.63	16.908	0.000
Varicella	<1 years	2	8.33	6	3.06	0.000	1.000
	1–6 years	13	54.17	107	54.59	13.733	0.000
	7–14 years	9	37.50	83	42.35	21.780	0.000
Scarlet fever	≤2 years	0	0.00	2	20.00		1.000^a^
	3–10 years	1	100.00	8	80.00	0.353	0.552
	11–14 years	0	0.00	0	0.00		

^a^Fisher's exact test.

**Table 5:** Analysis of pediatric infectious disease medical expenditures at Qingyang maternal and child health care hospital (Qingyang women's and children's hospital) during the COVID-19 pandemic and the post-pandemic period.

Disease name	2020–2022			2023–2025			Total		
	Number of cases	Total medical costs (¥)	Average medical cost per case (¥)	Number of cases	Total medical costs (¥)	Average medical cost per case (¥)	Number of cases	Total medical costs (¥)	Average medical cost per case (¥)
Influenza	0	0	0.00	1,224	2,746,750.98	2,244.08	1,224	2,746,750.98	2,244.08
Other infectious diarrheal diseases	3	20,697.15	6,899.05	675	2,434,379.05	3,606.49	678	2,455,076.2	3,621.06
Hand, foot, and mouth disease	1	2,984.59	2,984.59	301	95,745.61	318.09	302	98,730.2	326.92
Varicella	24	8,012.26	333.84	196	30,335.69	154.77	220	38,347.95	174.31
Pertussis	12	27,306.71	2,275.56	85	194,431.26	2,287.43	97	221,737.97	2,285.96
Mumps	6	1,050.9	175.15	5	3,972.5	794.50	11	5,023.4	456.67
Scarlet fever	1	2,985.97	2,985.97	10	20,799.6	2,079.96	11	23,785.57	2,162.32
Congenital syphilis	3	4,081.29	1,360.43	0	0	0.00	3	4,081.29	1,360.43
Hepatitis A	0	0	0.00	2	937.17	468.59	2	937.17	468.59
Measles	0	0	0.00	1	2,026.79	2,026.79	1	2,026.79	2,026.79
Other diseases	3	5,963.8	1,987.93	7	21,089.3	3,012.76	10	27,053.1	2,705.31
Total	53	73,082.67	1,378.92	2,506	5,550,467.95	2,214.87	2,559	5,623,550.62	2,197.56

USD amount = RMB amount/USD–CNY exchange rate.

### 3.4 Analysis of reported cases of key infectious diseases among children by age group

Surveillance data from Qingyang Maternal and Child Health Hospital indicate that no cases of influenza were reported among children between 2020 and 2022. In contrast, the number of reported cases for several pediatric infectious diseases increased significantly from 2023 to 2025 compared with the 2020–2022 period. Specifically: Pertussis cases rose significantly in both the 1–6-year-old group (*χ*^2^ = 4.246, *P* < 0.05) and the 7–14-year-old group (*χ*^2^ = 11.683, *P* < 0.05); Cases of other infectious diarrhea increased significantly among children aged 1–6 years (*χ*^2^ = 166.269, *P* < 0.05); Hand-foot-mouth disease (HFMD) incidence was significantly higher in both the 1–6-year-old group (*χ*^2^ = 85.124, *P* < 0.05) and the 7–14-year-old group (*χ*^2^ = 16.908, *P* < 0.05) than during the COVID-19 pandemic period; Varicella cases also showed a significant upward trend in both the 1–6-year-old group (*χ*^2^ = 13.733, *P* < 0.05) and the 7–14-year-old group (*χ*^2^ = 21.780, *P* < 0.05) (See Table 4).

### 3.5 Analysis of direct medical costs for childhood infectious diseases

The total direct medical cost was ¥5,623,550.62(RMB), following a trend broadly aligned with the number of cases. This cost was predominantly driven by influenza (¥2,746,750.98 RMB) and other infectious diarrheas (¥2,746,750.98 RMB), which together accounted for ¥5,201,827.18 RMB (92.5% of the total). The vast majority of these expenditures occurred in the post-pandemic period [¥5,550,467.95(RMB)], representing 98.70% of the overall cost; in contrast, costs incurred during the pandemic period totaled only ¥73,082.67 RMB (1.30% of the total). Notably, the average per-case medical cost was substantially higher in the post-pandemic period (¥2,214.87 RMB) than during the pandemic period (¥1,378.92 RMB). This discrepancy may reflect differences in data reporting practices, coding conventions, or rounding procedures—and therefore warrants further investigation and verification.

During the post-pandemic period, influenza [1,224 cases; ¥2,746,750.98(RMB)] and other infectious diarrheas [675 cases; ¥2,434,379.05(RMB)] were the two leading childhood infectious diseases in terms of both case volume and aggregate medical expenditure. In contrast, the average per-case medical costs for hand-foot-mouth disease [¥318.09 (RMB)], varicella [¥154.77(RMB)] and scarlet fever [2,079.96(RMB)] were lower in the post-pandemic period than during the pandemic period [¥2,984.59(RMB), ¥333.84(RMB) and ¥2,985.97(RMB), respectively]. Pertussis exhibited relatively stable average per-case costs across both periods [¥2,275.56(RMB) during the pandemic vs. ¥2,287.43(RMB) post-pandemic], remaining consistently high (See Table 5).

## 4 Discussion

Infectious diseases represent a significant threat to children's health. Common childhood infectious diseases are primarily transmitted via respiratory droplets and close contact. Non-pharmaceutical interventions (NPIs) implemented to curb the spread of coronavirus disease 2019 (COVID-19) have already exerted measurable effects on the epidemiology of other respiratory infections and fecal–oral–transmitted diseases (16–18). Analysis of infectious disease surveillance data for children aged ≤14 years collected at Qingyang Maternal and Child Health Care Hospital from 2020 to 2025 revealed a marked increase in the reported incidence rate—from 0.46 per thousand in 2020 to 10.32 per thousand in 2025—underscoring that infectious disease prevention and control remains a critical priority for maternal and child health specialty hospitals.

Our findings indicate that during 2020–2022, the majority of reported pediatric infectious disease cases occurred among children aged 3–6 years (37.74%), followed by those aged 0–≤2 years (33.96%) and school-aged children aged ≥7 years (28.30%). From 2023 to 2025, the age distribution shifted: children aged 0–≤2 years accounted for the largest proportion (46.89%), followed closely by those aged 3–6 years (37.15%), whereas the share of cases among children aged 7–14 years declined substantially (15.96%). Children aged <3 years—particularly those not enrolled in formal childcare settings (“scattered” or home-based children)—exhibit immature immune systems and have not yet developed consistent hygiene practices, rendering them highly susceptible to infectious diseases (19). Therefore, targeted health education for caregivers of scattered children is urgently needed. Schools constitute high-density congregate settings where infectious diseases readily propagate and amplify (20); thus, strengthening daily morning and midday health checks, tracking absences due to illness, and ensuring timely catch-up vaccination for missed doses are essential preventive measures. The observed decline in infectious disease reporting among 7–14-year-olds post-pandemic likely reflects both the durable immunity conferred by routine vaccination and improved hygiene behaviors; nevertheless, continued vigilance against vaccine-preventable respiratory infections—including influenza and varicella—as well as emerging concerns regarding sexually transmitted infections (STIs) remains warranted.

Notably, no cases of influenza were reported among children aged ≤14 years at Qingyang Maternal and Child Health Care Hospital during the pandemic period (2020–2022), and reporting rates for other infectious diarrheas and hand-foot-mouth disease (HFMD) remained low. This suppression is plausibly attributable to widespread NPI implementation—including mask-wearing, frequent handwashing, maintenance of ≥1-meter interpersonal distancing, school closures/remote learning, and travel restrictions—which collectively reduced person-to-person transmission opportunities (21). Additionally, heightened fear of SARS-CoV-2 infection likely discouraged non-urgent healthcare visits, contributing to underreporting (22). In contrast, a dramatic rebound occurred in the post-pandemic period (2023–2025), with overall reporting rates surging to over tenfold those observed during 2020–2022—reaching their highest level in 2025. This pattern aligns with global observations of “rebound epidemics” of respiratory and contact-transmitted infections following pandemic-related NPI relaxation (16). Among the top three conditions driving this resurgence were influenza (48.72%), other infectious diarrheas (26.94%), and HFMD (12.01%). Possibly due to the implementation of non-pharmaceutical interventions during the pandemic (23, 24), children's exposure to pathogenic microorganisms was reduced, leading to insufficient sustained immunostimulation by these pathogens, a decline in population-level immunity, and increased susceptibility (25). The magnitude of rebound differed markedly by pathogen: influenza exhibited a particularly strong resurgence, likely attributable to its high antigenic drift rate and the rapid waning of humoral immunity following natural infection or inactivated vaccination (26), whereas measles and mumps—which are targeted by live-attenuated vaccines that confer more durable protection—showed minimal post-pandemic increase. Furthermore, natural infection typically elicits broader and more sustained cellular and humoral immune memory than inactivated vaccines, which may partly explain why immunity debt disproportionately affected pathogens for which vaccine-induced protection is less robust or shorter-lived (27). As societal activities normalized, cumulative exposure to pathogens intensified—especially in congregate settings such as schools and kindergartens—triggering rapid case escalation. Moreover, pathogen-specific transmission dynamics differ markedly: influenza virus and enteric pathogens such as norovirus and rotavirus possess exceptionally high transmissibility; consequently, even modest reductions in NPI adherence can precipitate explosive outbreaks (28). Pertussis incidence increased markedly in the post-pandemic period—a trend consistent with national surveillance data (29, 30). Moreover, immunity induced by the acellular pertussis vaccine wanes over time (31), and suboptimal dosing of the diphtheria–tetanus–acellular pertussis (DTaP) vaccine further compromises immunogenicity. These findings underscore the need to strengthen DTaP booster immunization programs to enhance protective immunity in children (32, 33) and suboptimal dosing of the diphtheria–tetanus–acellular pertussis (DTaP) vaccine further compromises immunogenicity. These findings underscore the need to strengthen DTaP booster immunization programs to enhance protective immunity in children.

The epidemiological characteristics of infectious diseases and associated healthcare expenditures in this population have undergone significant changes. In the post-pandemic period, the outpatient burden of infectious diseases among children aged ≤14 years markedly increased, with total healthcare expenditures amounting to ¥5,623,550.62(RMB), particularly driven by influenza and other infectious diarrheas, which accounted for ¥5,201,827.18(RMB). Notably, the average per-case healthcare costs for other infectious diarrheas, hand-foot-mouth disease (HFMD), and varicella were lower in the post-pandemic period than during the pandemic, this may be associated with changes in disease severity or alterations in healthcare-seeking behavior. A promising finding is that average per-case costs for other infectious diarrheas, HFMD, and varicella declined after the pandemic compared with those observed during the pandemic, this may be attributable to the fact that, during the pandemic, only severe cases were likely to seek hospital-based care—thereby incurring higher per-case costs. In contrast, in the post-pandemic period, the surge in mild and self-limiting illnesses (particularly influenza and hand-foot-mouth disease), coupled with improved access to healthcare services, has naturally reduced the average cost per case—even as total healthcare expenditures have risen sharply. However, pertussis consistently incurred high average costs across both periods, underscoring its clinically complex management and prolonged treatment course—highlighting the need for sustained attention to its economic burden (34).

### 4.1 Limitations

First, the data were derived exclusively from maternal and child health institutions in Qingyang City, primarily reflecting the characteristics of children who sought medical care; therefore, these data may not fully represent the complete epidemiological profile of infectious diseases among all children in the city. Second, the cost analysis encompassed only direct medical expenses and excluded indirect household-level costs—including, but not limited to, caregiver absenteeism (e.g., lost wages) and transportation expenses—potentially leading to an underestimation of the overall economic burden of these diseases. Future research will aim to comprehensively quantify the disease burden of pediatric infectious diseases, incorporating both direct and indirect costs.

### 4.2 In summary

In the post-pandemic era, pediatric infectious disease prevention and control face new challenges. The disease spectrum has shifted from being predominantly composed of vaccine-preventable diseases toward those characterized by high transmissibility and epidemic potential—especially influenza and infectious diarrheas. Accordingly, we propose the following evidence-informed recommendations: (1) Strengthen infectious disease surveillance and early-warning systems in schools and childcare facilities, particularly during peak influenza seasons and high-incidence periods of diarrheal illnesses; (2) Actively promote the uptake of non-immunization program vaccines—including influenza, rotavirus, and HFMD vaccines—to establish broader population-level immunity; and (3) Optimize clinical diagnostic and treatment pathways to maintain therapeutic efficacy while containing healthcare expenditures, thereby alleviating economic burdens on both society and families.

## Data Availability

The original contributions presented in the study are included in the article/Supplementary Material, further inquiries can be directed to the corresponding author.
